# Defocus incorporated multiple segments (DIMS) spectacle lenses increase the choroidal thickness: a two-year randomized clinical trial

**DOI:** 10.1186/s40662-023-00356-z

**Published:** 2023-09-15

**Authors:** Rachel Ka Man Chun, Hanyu Zhang, Zhengji Liu, Dennis Yan Yin Tse, Yongjin Zhou, Carly Siu Yin Lam, Chi Ho To

**Affiliations:** 1 Centre for Myopia Research, School of Optometry, The Hong Kong Polytechnic University, Kowloon, Hong Kong, China; 2 Research Centre for SHARP Vision (RCSV), The Hong Kong Polytechnic University, Kowloon, Hong Kong, China; 3 Centre for Eye and Vision Research (CEVR), 17W Hong Kong Science Park, Shatin, Hong Kong, China; 4 School of Medicine, Nankai University, Tianjin, China; 5 School of Biomedical Engineering, Department of Medical Electronics, Shenzhen University, Shenzhen, China

**Keywords:** Choroidal thickness, Optical defocus, DIMS, Myopia

## Abstract

**Background:**

Myopia control interventions, such as defocus incorporated multiple segments (DIMS) spectacle lenses, have been adopted in school-aged children to reduce the prevalence of myopia and its complications. This study aimed to investigate the effect of DIMS spectacle lenses on subfoveal choroidal thickness (SfChT) over a period of two years, as the choroidal response to myopic control is a crucial factor in exploring its potential effect on predicting myopia progression.

**Methods:**

This study involved a secondary analysis of our previous randomized clinical trial. Myopic school-aged children aged 8–13 years were recruited in a two-year study investigating the effect of DIMS spectacle lenses on myopia progression. The treated group received DIMS spectacle lenses (n = 78), while the control group was treated with a pair of single vision (SV) spectacle lenses (n = 80). SfChT was monitored at 1 week, 1, 3, 6, 12, 18 and 24 months post lens wear using spectral-domain optical coherence tomography and a custom made auto-segmentation algorithm utilizing convolutional neural networks.

**Results:**

SfChT increased significantly after one week of DIMS spectacle lens wear compared to those wearing SV spectacle lenses (adjusted mean change relative to baseline ± SEM at one week; DIMS *vs.* SV, 6.75 ± 1.52 µm *vs*. − 3.17 ± 1.48 µm; *P* < 0.0001, general linear model). The thickness of choroid increased to 13.64 ± 2.62 µm after 12 months of DIMS lens wear while the choroid thinned in SV group (− 9.46 ± 2.55 µm). Choroidal changes demonstrated a significant negative association with axial elongation over two years in both the DIMS and SV groups. Choroidal change at three months significantly predicted the changes in AL at 12 months after controlling the effect of age and gender.

**Conclusions:**

Our study demonstrated a significant choroidal thickening in response to myopic defocus incorporated in a spectacle lens after one week of lens wear, sustained over the two-year study period. The results suggested that choroidal changes at three months may help predict changes in axial length after one year.

*Trial registration* ClinicalTrials.gov. Myopia control with the multi-segment lens. NCT02206217. Registered 29 July 2014, https://clinicaltrials.gov/ct2/show/study/NCT02206217

## Background

The prevalence of myopia remained high in the past few decades, particularly in East Asian regions [1]. It is anticipated that approximately half of the world population will be affected by myopia in 2050, with 10% of them suffering from high myopia [2]. High myopia is strongly associated with ocular pathologies such as glaucoma and retinal degenerations [3]. These sight threatening diseases significantly impede daily activities and also increase the financial burden to public health system [4, 5]. Controlling the progression of myopia in school-aged children is necessary to decrease the prevalence of high myopia and minimize the risk of associated complications in adulthood. There are various myopic control interventions that can effectively retard the progression for school-aged children. These interventions include low dose atropine [6, 7], repeated low-intensity red light therapy [8] and optical interventions such as orthokeratology, contact lenses and spectacle lenses [9, 10].

Myopic defocus is widely applied in the optical interventions such as defocus incorporated soft contact (DISC) lenses and defocus incorporated multiple segments (DIMS) spectacle lenses [11, 12]. Myopic defocus is formed when a positive powered lens is placed in front of the full corrected eyes. The magnitude of myopic defocus incorporated in DISC and DIMS lenses were + 2.5 D and + 3.5 D, respectively. School-aged children who wore DISC lenses for over eight hours have shown a 60% reduction in myopia progression [11], while a DIMS study has also demonstrated a similar retardation in myopia progression among school-aged children in a two-year randomized controlled trial [12].

Although the progression of myopia can be effectively retarded, the exact mechanism of myopia development is not fully understood. The choroid has been suggested to play a role in modulating the eye growth in both animal models and humans [13, 14]. In chicks, the choroidal thickness and choroidal blood flow increased significantly when myopic defocus was imposed for several hours [15, 16]. These choroidal changes were rapid and robust. With the advent of optical coherence tomography (OCT), the choroidal thickness could be continuously monitored in both adults and school-aged children in a non-invasive manner [17]. Our previous findings demonstrated a significant alteration in the subfoveal choroidal thickness (SfChT) in school-aged children after a two-hour exposure to optical defocus [18]. The SfChT is defined as the measurement of choroidal thickness at the center of foveola [19]. However, the impact of long-term exposure to myopic defocus through DIMS lenses on the choroidal thickness of school-aged children remains uncertain.

In view of the above considerations, we aimed at investigating the long-term effect of myopic defocus that is incorporated in spectacle lenses on SfChT of school-aged children in a two-year randomized controlled study. This study was a secondary analysis of our previous randomized controlled trial that investigated the effect of DIMS lenses on myopia progression in 8–13-year-old children [12].

## Methods

### Subject participants

One hundred and eighty three school-aged children aged 8–13 years in Hong Kong China were recruited to participate in a two-year prospective, randomized and double-masked clinical trial to investigate the effect of DIMS lens wear on controlling myopia progression in school-aged children [12]. All subjects and their parents or guardians signed an informed assent and consent. The study protocol was approved by Institutional Review Board of The Hong Kong Polytechnic University (HSEARS20140630003) and was conducted in accordance with the ethical principles of the Declaration of Helsinki.

Both eyes were myopic with at least − 1.00 D to − 5.00 D in spherical equivalent. The anisometropia were no more than − 1.50 D. Their binocular vision, color vision and ocular health were normal. They had no previous use of myopic control intervention such as atropine, orthokeratology, specific spectacles or contact lenses. Eligible subjects were randomly allocated into treatment group, that was DIMS lens wear, or control group that was corrected with the single vision (SV) spectacle lenses. The screening procedures and randomization were mentioned in our previous study [12]. One hundred and sixty subjects aged between 8 and 13 years completed the two-year randomized clinical trial, in which 79 and 81 subjects were in DIMS and SV groups, respectively [8].

### Study procedures and data collection

Children in both DIMS and SV lens groups were required to wear the assigned spectacle lenses in full-time mode, except while taking showers and sleeping. Both the subjects and their parents were masked from grouping as in our previous publication [12]. Subjects in both groups were required to have follow-up visits at 1 week, 1 month, 3 months, 6, 12, 18 and 24 months post lens wear.

Macular OCT images from both eyes, acquired by a spectral-domain OCT (Spectralis SD OCT, Heidelberg Engineering Inc., Heidelberg, Germany), were collected at baseline. OCT images were collected at a similar time during subsequent follow-ups to minimize the effect of diurnal variation on the choroidal thickness. All OCT images were acquired without the use of cycloplegia. Three horizontal and three vertical line scans with averaging 100 B-scan images and scanning angle of 30 degrees were collected in each eye. Enhanced depth imaging mode was adopted for image acquisition to capture the boundary between choroid and sclera. OCT images obtained at baseline were set as a reference, and the subsequent data was collected based on that reference.

### Automated choroidal segmentation using convolutional neural networks (CNN)

OCT images were extracted from the built-in software of Heidelberg spectral-domain OCT for further analysis. We developed a fully automated segmentation method/model to extract the SfChT in OCT images using convolutional neural networks (CNNs). An U-Net [20] with batch normalization and a ResNet-101 [21] were employed in this custom made method. Both networks were modified by replacing the bottleneck with strip pooling block [22] so as to increase the receptive fields of the network in horizontal and vertical directions. A late fusion method, which involved averaging the prediction scores of both models' outputs, was also employed to enhance the overall segmentation accuracy and reduce the impact of individual model limitations.

The models were then trained using the OCT images collected over the study. Specifically, 224 OCT images were randomly selected for model training while 128 images were designated for evaluation purposes.

### Training and evaluation metrics

During the training procedure, Adam optimizer [23] and dice loss were chosen as the optimizer and loss functions, respectively. Dice loss was specifically selected to optimize the dice score (DSC), which is a metric to evaluate the accuracy of a segmentation algorithm [24]. Online data augmentation techniques, such as random rotations (± 10°), random horizontal flipping, and random color jitter (brightness, contrast, and saturation), were also implemented during the training process to enhance model generalization. DSC and average symmetric surface distance (ASSD) were chosen for evaluating the segmentation performance, and their equations are as follow:

$$\text{DSC(X, Y)}=\frac{2\left|X\cap Y\right|}{\left|X\right|+\left|Y\right|}$$

X represented the binary image of the segmentation result, Y represented the ground truth annotation.

$$ASSD=\frac{1}{N}{\sum }_{i=1}^{N}\left({d}_{i}+{d}_{i}^{*}\right)$$

where *N* was the number of surface points on the segmented surface, ${d}_{i}$ was the distance between the *i-*th surface point on the segmented surface and its nearest surface point on the ground truth surface, and ${d}_{i}^{*}$ was the distance between the *i*-th surface point on the ground truth surface and its nearest surface point on the segmented surface.

Apart from DSC and ASSD as the performance metrics, another 155 images collected from the baseline were used for validation. Choroidal thickness was manually measured using the built-in software of Heidelberg spectral-domain OCT by an independent grader. The measurements were then compared between manual and automated approach using linear regression.

### Post-processing and identification of fovea

**Fig. 1:**
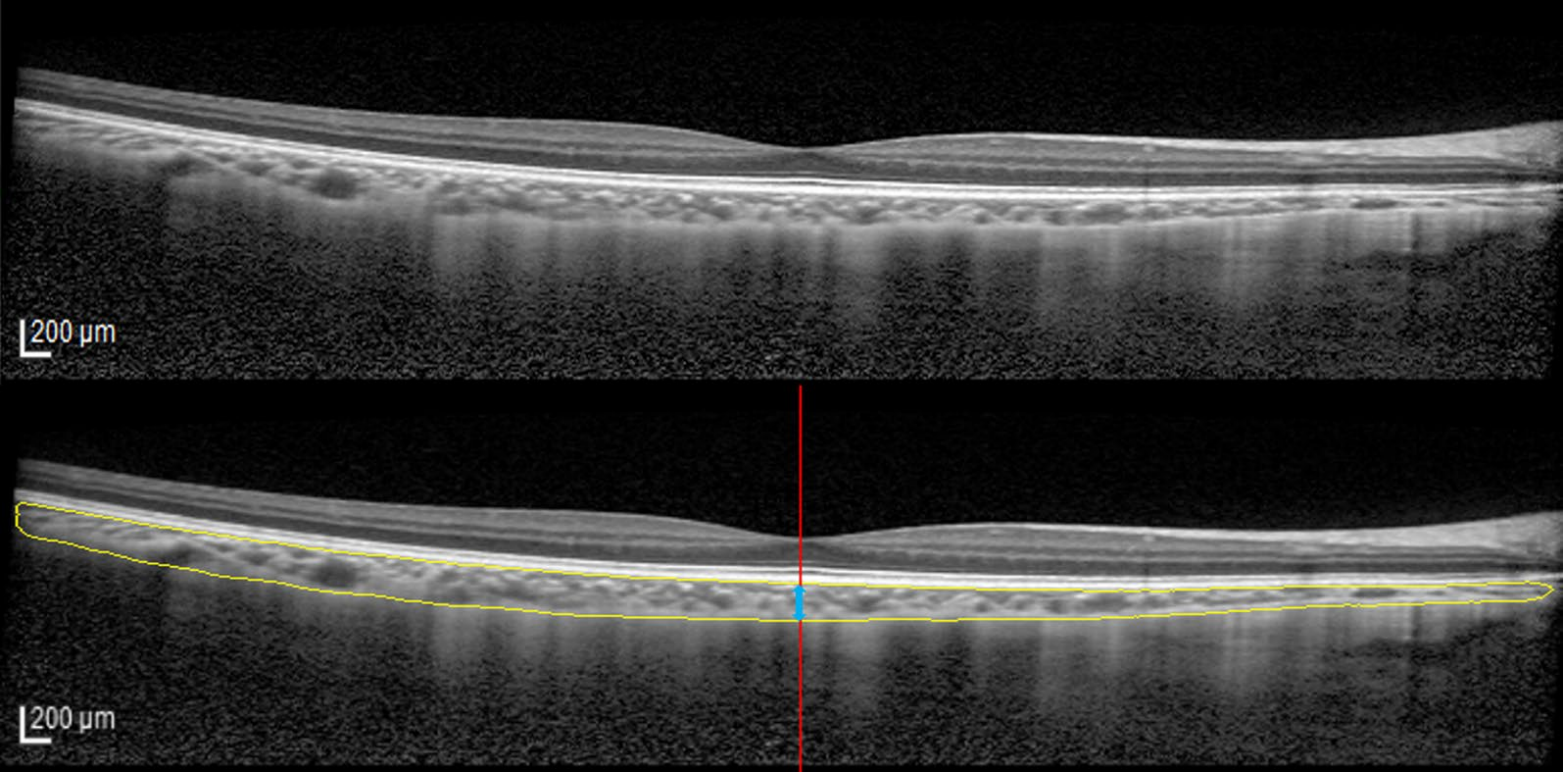
Representative optical coherence tomography (OCT) images showing the performance of the current automated segmentation model in predicting the choroidal layer. The original OCT image is displayed at the top, with the choroidal layer being highlighted in yellow in the bottom image. Red line denotes the location of the fovea. Blue arrow indicates the subfoveal choroidal thickness

After training the models, the outputs were binarized using a threshold of 0.5 to separate the background and choroid in OCT images. Choroid in OCT images were defined as an area between retinal pigmented epithelium and the chorio-scleral boundary. A maximally connected domain approach was applied to eliminate false-positive predictions located outside the choroid in the OCT image. Subsequently, an automatic identification of fovea was applied before measuring choroidal thickness at the subfoveal region. To find the location of the fovea, 25 pixels at the middle of the image were selected and Otsu binary segmentation [25] was perform to segment the retinal layer from OCT images. The location with the thinnest retina was defined as the location of fovea. Choroidal thickness at the subfoveal region was then extracted at the point where the retinal thickness was the thinnest (Fig. 1). SfChT was averaged from three horizontal and three vertical line scans.

Other than OCT images, cycloplegic refraction and axial length (AL) were measured every six months for two years. Cycloplegia were carried out by instilling one to two drops of cyclopentolate hydrochloride and refractions were measured using an open field autorefractor (Shin-Nippon NVision-K5001). AL was measured by partial coherence interferometry using the IOL Master 500 (Carl Zeiss, Germany). Five readings of autorefraction and AL for each eye were averaged for data analysis.

### Outcome variables

OCT images were collected in all follow-up visits while refraction and AL under cycloplegia were only measured at baseline and at six-month intervals. The primary outcome was choroidal thickness at the subfoveal region while the secondary outcomes were myopia progression and axial elongation. Myopia progression was defined as the changes in cycloplegic spherical equivalent refraction between baseline and at six-month intervals. Axial elongation was the difference of AL between baseline and subsequent six-month follow up for two years.

### Statistical analysis

Data of right eyes were used for data analysis (SPSS software version 26; SPSS Inc., Chicago, IL). Baseline measurements such as age, gender ratio, AL, spherical equivalent refractive errors (SER) and SfChT between DIMS treatment group and SV group were compared using unpaired t-test.

Changes in SfChT, SER and AL at different time points relative to the baseline were calculated. Mixed between-within subjects analysis of variance was used to compare changes in SfChT between DIMS group and SV group during the experimental period, with the assumption of homogeneity of covariate regression slopes between groups. The baseline parameter was adjusted as a covariate if the baseline measurement was significantly different between groups.

Association between changes in SfChT and SER or AL was examined using Pearson’s coefficient analysis. Hierarchical multiple regression was applied to explore if the short-term choroidal changes could predict the change in axial elongation after 12 months. A *P* value of < 0.05 was considered as statistically significant.

## Results

### Performance metrics of models involved in auto-segmentation of choroid

**Fig. 2:**
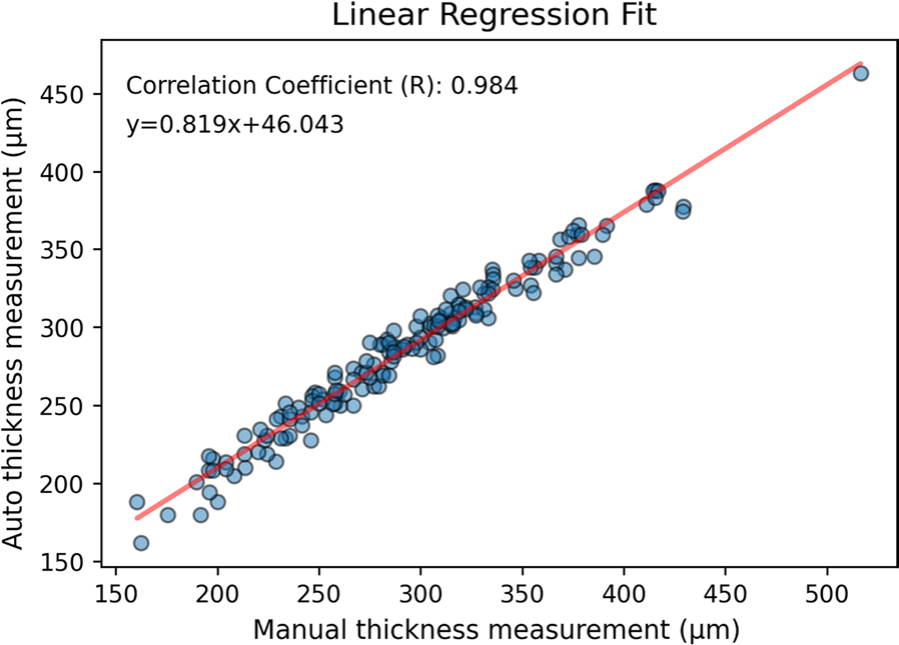
The relationship between the choroidal thickness measured by manual and automatic segmentation

Performance metrics including DSC and ASSD in different models were shown in Table 1. All models in the study showed promising mean DSC (over 94). In terms of ASSD, the fusion model exhibited a better segmentation performance with the lowest mean ASSD value of 1.58 ± 1.08. The relationship between choroidal thickness measured by manual and automated measurement was shown in Fig. 2. Our findings revealed a strong and statistically significant correlation between both measurements (R = 0.984, *P* < 0.0005), indicating a high level of similarity between the choroidal thickness measurement obtained from the automated segmentation algorithm and the manual measurement.

**Table 1:** Performance metrics including dice score (DSC) and average symmetric surface distance (ASSD) in different models of the current auto-segmentation algorithm

Model	DSC (mean ± SD)	Maximum value of DSC	ASSD (mean ± SD)
U-Net	94.79 ± 3.05	98.31	1.75 ± 1.40
ResNet-101 with SP block	94.54 ± 3.15	97.92	1.73 ± 1.15
U-Net with SP block	95.01 ± 2.88	98.32	1.60 ± 1.07
Fusion model	94.99 ± 2.94	98.31	1.58 ± 1.08

### Baseline characteristics

Two subjects (one from DIMS while the other from SV group) were excluded because of a missing baseline choroidal thickness measurement. At baseline, no statistically significant difference was found in age, gender ratio, cycloplegic SER and AL between the two groups, except baseline SfChT. SfChT in the DIMS group were significantly thicker than those in the SV group (mean ± SD; DIMS vs. SV; 280.95 ± 54.30 µm vs. 259.53 ± 49.36 µm, Table 2, mean difference = 21.95 ± 8.29 µm, *P* = 0.009, unpaired t-test). There was no age effect on the choroidal thickness at baseline (*P* = 0.107, one-way ANOVA, Table 3). The choroidal thickness in 9-year-old subjects in the DIMS group was significantly thicker than those in the SV group (*P* = 0.04, unpaired t-test). Therefore, the results from a general linear model were adjusted for baseline SfChT as a covariate.

**Table 2:** Demographic data of all subjects

Demographic data	Defocused incorporated multiple segments spectacle lenses (n = 78) Mean (SD)	Single vision spectacle lenses (n = 80) Mean (SD)
Age at enrolment (years)	10.20 (1.47)	10.00 (1.45)
Gender		
Male, % (n)	58.2 (46)	54.3 (43)
Female, % (n)	41.8 (32)	45.7 (37)
Cycloplegic autorefraction in SER (Dioptres)	− 2.97 (0.97)	− 2.76 (0.96)
Axial length (mm)	24.70 (0.82)	24.60 (0.83)
Subfoveal choroidal thickness (µm)	280.95 (54.30)	259.53 (49.36) *P* = 0.009, unpaired t-test

*SER* = spherical equivalent refraction; *SD =* standard deviation

**Table 3:** Mean subfoveal choroidal thickness in different age groups at baseline

Age (years)	Mean (SD)	DIMS group	SV group	Significance
8	259.98 (57.80)	274.42 (59.61) n = 8	249.48 (60.50) n = 11	*P* = 0.384
9	279.24 (52.82)	294.26 (52.61) n = 23	261.97 (48.72) n = 20	*P* = 0.044
10	275.97 (46.97)	278.00 (48.28) n = 20	273.42 (46.71) n = 16	*P* = 0.777
11	260.32 (57.51)	271.66 (62.69) n = 13	246.91 (50.25) n = 11	*P* = 0.304
12	253.35 (50.00)	265.45 (57.40) n = 11	245.03 (44.21) n = 16	*P* = 0.306
13	300.71 (39.91)	313.06 (51.88) n = 3	294.54 (36.53) n = 6	*P* = 0.548

*DIMS* = defocus incorporated multiple segments; *SV* = single vision; *SD* = standard deviation

### Changes in subfoveal choroidal thickness

The changes in SfChT at each time point were compared using mixed between-within subjects analysis of variance with the adjustment for the baseline choroidal thickness. There was no significant main effect for time, Wilks’ Lambda = 0.99, F (6, 137) = 0.354, *P* = 0.91, partial era squared = 0.015. However, a significant interaction between groups and time was found (Wilks’ Lambda = 0.86, F (6, 137) = 3.686, *P* = 0.002, partial era squared = 0.139).

**Fig. 3:**
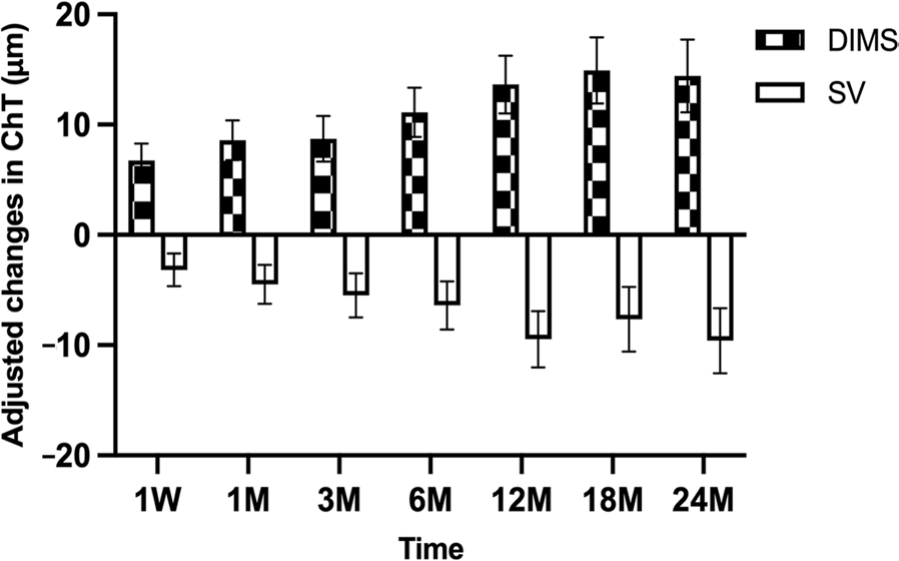
Effect of defocus incorporated multiple segments (DIMS) and single vision (SV) spectacle lenses on the subfoveal choroidal thickness (ChT) over two years. Relative changes in ChT to baseline were calculated and adjusted at 1 week (1W), 1 month (1 M), 3 M, 6 M, 12 M, 18 M and 24 M post lens wear. Data are shown as mean ± SEM. Choroidal thickness increased significantly after the DIMS lens wear at all time points (*P* < 0.0005, mixed repeated measures ANOVA)

The main effect comparing the changes in choroidal thickness in DIMS and SV groups was significant [*P* < 0.005, F (1, 142) = 50.08, partial eta squared = 0.261] suggesting that there was a significant effect of DIMS lenses on choroidal thickness changes over the study (Fig. 3). There was a significant thickening of SfChT in response to DIMS lens wear after one week. The thickening increased over the first year and sustained at 18 months onwards. Conversely, the SfChT in SV group thinned continuously over two years of study (Fig. 3).

### Relationship between changes in choroidal thickness and myopia progression

**Fig. 4:**
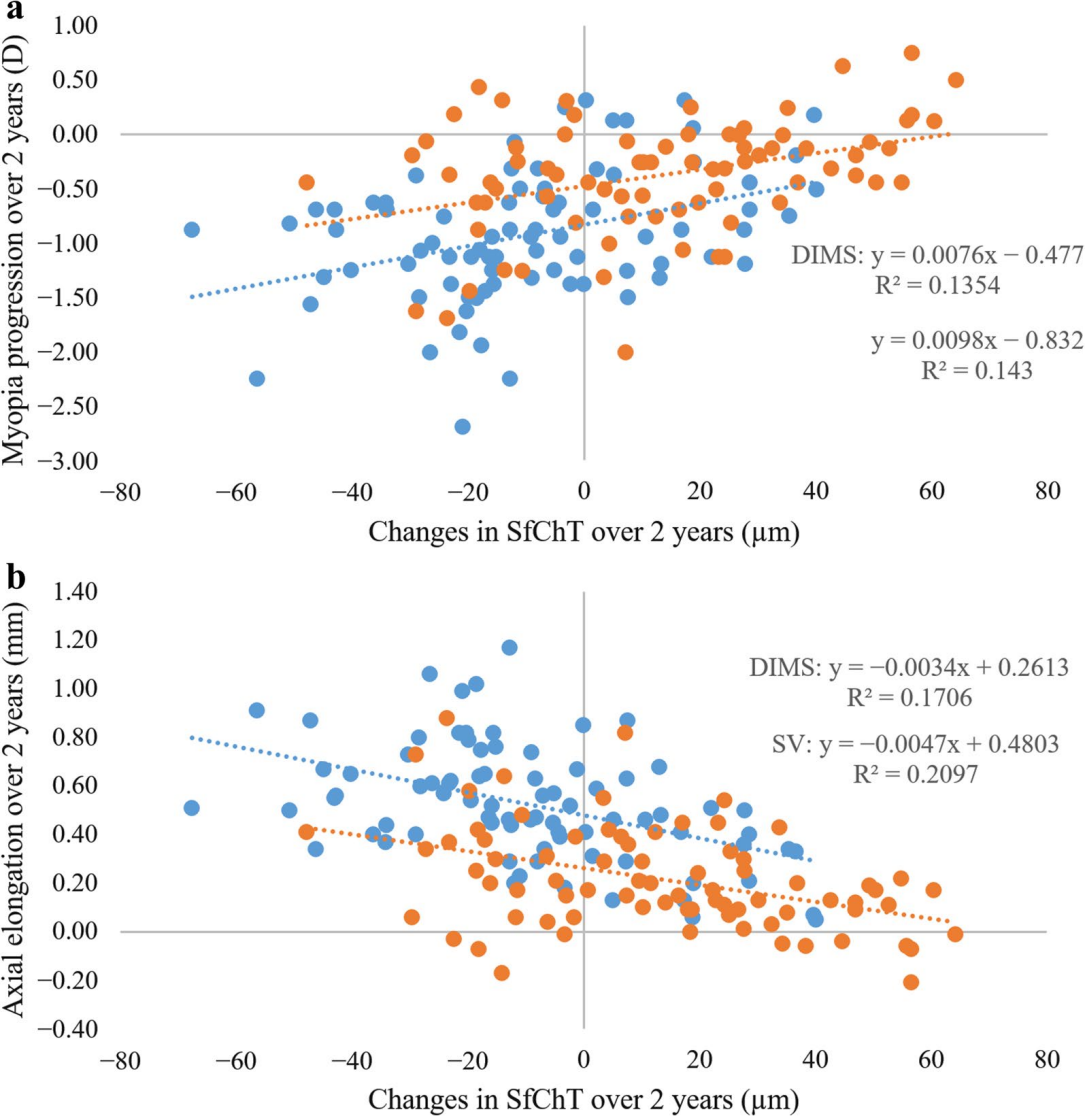
Correlation between changes in subfoveal choroidal thickness (SfChT) and myopia progression (**a**) and axial elongation (**b**) over two years

There was a negative association between changes in SfChT and axial elongation over two years in DIMS and SV group (Pearson coefficient; DIMS *vs*. SV group: − 0.41, *P* < 0.0001 vs. − 0.46, *P* < 0.0001, Fig. 4). Conversely, changes in SfChT were positively associated with the progression of myopia in both groups (Pearson coefficient; DIMS vs. SV group; 0.37, *P* < 0.001 vs. 0.38, *P* < 0.001, Fig. 4). These findings indicated that the magnitude of choroidal thickening was associated with the myopia progression with respect to the changes in spherical equivalent or AL. As the magnitude of choroidal thickening increased, myopia progression decreased over two years. The different slopes observed between the DIMS and SV groups suggest a difference in the strength of the relationship between choroidal changes and axial elongation in each group. Comparison of the slopes depicted in Fig. 4b indicates that changes in choroidal thickness may account for 27.7% of the observed axial elongation.

### Investigating the potential of short-term changes in choroidal thickness to predict axial elongation

Optical stimulation with either myopic or hyperopic defocus contributed to the changes in choroidal thickness in both animal models and humans [26]. Based on this observation, the short-term choroidal responses and axial elongation in both DIMS and SV group were used to explore their relationship. Hierarchical multiple regression was used to assess the ability of short-term choroidal change (1 week, 1 month and 3 month) to predict AL changes at 12 months after controlling for the influence of age and gender. Age and gender explained 9% of variance in the 12-month AL changes (Table 4). After inputting the choroidal thickness changes at 1 week, 1 month and 3 months, only the choroidal changes at three months significantly explained 12.6% of variance in changes of AL at 12 months after controlling for the influence of age and gender (R square = 0.126, *P* < 0.005). In the final model, age had a higher beta value (beta = − 0.30, *P* < 0.005) than change at 3 months (beta = − 0.20, *P* = 0.01, Table 4).

**Table 4:** Multiple regression models for myopia progression in terms of axial elongation at 12 months

Parameter	Model summary	Standardized coefficients beta	Significance
Model 1	R square = 0.086 (*P* = 0.001*)		
Age		− 0.289	< 0.005*
Gender		− 0.038	0.625
Model 2	R square = 0.105 (*P* = 0.001*)		
Age		− 0.297	< 0.005*
Gender		− 0.21	0.788
ChT changes at 1 week		− 0.140	0.073
Model 2	R square = 0.092 (*P* = 0.002*)		
Age		− 0.293	< 0.005*
Gender		− 0.037	0.629
ChT changes at 1 month		− 0.081	0.294
Model 2	R square = 0.126 (*P* < 0.005*)		
Age		− 0.299	< 0.005*
Gender		− 0.021	0.788
ChT changes at 3 months		− 0.203	0.010*

Asterisk (*) indicated that the variable had a significant unique contribution to the prediction of the dependent variable

*ChT* = choroidal thickness

## Discussion

This study presents the first evidence of the effect of DIMS lenses on SfChT in school-aged children over a two-year randomized controlled trial. Both short-term and long-term effects of DIMS lenses on choroidal thickness were revealed. The choroid thickened significantly in response to one-week DIMS lens wear compared to those wearing SV lenses as control. The thickness further increased when the school-aged children continued the DIMS lens wear until 18 months of lens wear. The thickening was stabilized and sustained over the second year of lens wear (Fig. 3).

The magnitude of choroidal thickening found in the current study was relatively smaller than those in animal models such as chicks [27, 28]. The thickening in response to myopic defocus was fast and robust in chicks [13]. An exposure of + 10 D myopic defocus for 10 min resulted in choroidal expansion that accounted for approximately 17% of its thickness in chicks [16]. The discrepancy could be due to the structural difference in the chick’s choroid which has large lacunae and may facilitate more drastic thickening [29, 30]. Also, the paradigm in delivering myopic defocus in chicks and human was different. Chicks received a full-field + 10 D optical lens while myopic defocus was delivered via multiple segments at periphery surrounding the central zone for distance refractive correction in school-aged children wearing DIMS lenses [12]. Whether there is an interaction between the central distant zone and peripheral areas with myopic defocus on choroidal response awaits further study.

The choroidal response to myopic defocus in the current study was comparable to those observed in school-aged children from our previous study [18]. Both studies showed a choroidal thickening to myopic defocus. Our findings demonstrated that the SfChT of school-aged children increased by 2.4% after wearing DIMS lenses for one week (adjusted mean change/baseline choroidal thickness; 6.75 µm/280.95 µm × 100% = 2.4%). The magnitude of the increase in choroidal thickness was similar to our previous findings on myopic school-aged children who demonstrated a relative thickening (around 3%) in the choroid when + 3 D myopic defocus was imposed for 1–2 h [18]. Although the magnitude of the choroidal thickening was comparable between two studies, the duration of exposure to myopic defocus differed. Unlike the previous study [18], which only examined the choroidal thickness changes after 1–2 h of + 3 D myopic defocus, the current study did not monitor the short-term response of choroidal thickness after one to two hours of DIMS lens wear. Consequently, it remained unclear whether the choroid thickens immediately after 1–2 h of DIMS lens wear. Thus, further investigation is necessary to determine the choroidal response to short-term exposure to DIMS lenses.

The changes in choroidal thickening in DIMS wearers over time were different from those school-aged children who received the treatment of orthokeratology in previous studies [31, 32]. The magnitude of choroidal thickening after wearing DIMS lenses for one month was smaller than those treated with orthokeratology for three weeks (one-month DIMS lens vs. three-week orthokeratology; 8.58 µm vs. 21.8 µm) [31]. However, a more recent study demonstrated a smaller increase in choroidal thickening after four weeks of orthokeratology wear. There was a 9-µm increase at one-week of lens wear and the thickening was maximum at the first week of orthokeratology [33]. Interestingly, the increase in choroidal thickness was transient and it then decreased gradually and went back to baseline after four weeks of orthokeratology [33]. The changes in the choroidal thickening over time were different in school-aged children wearing DIMS lenses, where the thickening continued to increase and was sustained throughout the two-year study period. Both DIMS lens and orthokeratology retard myopia progression by imposing myopic defocus [34], however, the amount of myopic defocus induced by orthokeratology varies between individuals [35]. Variations in the lens design and fitting of orthokeratology may lead to differences in the amount of myopic defocus induced [36] which could potentially account for the observed discrepancy in choroidal responses between orthokeratology and DIMS lenses.

Our results showed a sustained thickening of choroid, which differed from a recent study that investigated the effect of myopic control spectacle lenses with aspherical lenslets on choroidal thickness in school-aged children [37]. The choroidal thickness increased after six months of myopic control spectacle lenses with aspherical lenslets, however, the thickening only lasted for the first year, and the choroidal thickness returned to the baseline in the second year of the randomized controlled trial [37]. Conversely, the choroidal thickening induced by DIMS lenses in the current study was sustained over two years of study. The discrepancy could be due to the gradual adaptation of the retina to the signals elicited by the aspherical lenslets, which may result in a reduction of the effect of choroidal thickening over time [37].

In contrast, when comparing the choroidal response to low dose atropine, choroidal thickening to both atropine and DIMS lens remained stable over two years in randomized clinical trials [38, 39]. The thickening in response to atropine was shown in a dose-dependent manner [38]. Higher concentrations of atropine such as 0.05% induced a greater increase in choroidal thickness than lower concentrations (0.01%) [38]. However, our study could not reveal any dose-dependent relationship between magnitude of myopic defocus and choroidal thickness changes. This was attributed to the limited degree of myopic defocus (+ 3.5 D) imposed by DIMS lenses.

The underlying mechanism of choroidal thickening in response to DIMS lens is currently unclear. Intrinsic choroidal neurons are suggested to play a role in modulating changes in choroidal thickness and blood flow during eye growth [40]. The human choroid contains both vascular and non-vascular smooth muscle cells that innervated by intrinsic choroidal neurons [41, 42]. These intrinsic choroidal neurons could regulate the choroidal blood flow by producing vasodilators such as nitric oxide and vasoactive intestinal polypeptide on smooth muscle cells [13, 40]. As a result, the choroidal blood flow increases with the thickening of choroid.

This study found a significant increase in choroidal thickness at the subfoveal region after a week of DIMS lens wear, compared to children who received SV lenses as controls. Although short-term changes in SfChT were not fully predictive of axial elongation changes at 12 months, this does not preclude the potential usefulness of monitoring choroidal changes in the future. Whether changes in choroidal thickness at specific regions in the posterior eye, such as the periphery or specific quadrants, play a more significant role in prediction remains unclear. Given that myopia development is a multifactorial process [1], predicting its progression is likely to require consideration of multiple parameters such as parental myopia and age of myopia onset. Further investigation is needed, utilizing swept source OCT, which provides higher resolution of the chorio-scleral boundary, to assess the potential predictive capability of choroidal thickness at the periphery in long-term axial elongation.

There were several limitations in the current study. The baseline choroidal thickness of the school-aged children who received DIMS lenses were significantly thicker than those received SV lenses as control. Despite controlling for this covariate during data analysis, the possibility remains that the thicker choroidal thickness observed in the DIMS group at baseline may have contributed to the greater responsiveness to DIMS lenses. Apart from the baseline measurement, only horizontal and vertical line scans of cross-sectional OCT images were obtained rather than the volume scan during the data collection. Furthermore, only changes in choroidal thickness at the subfoveal region were reported. The current study failed to indicate if there were changes in volume of choroid across the posterior pole in response to DIMS lenses. Therefore, it is unclear if there was a regional difference in choroidal response to myopic defocus induced by DIMS lenses. The use of spectral-domain OCT in monitoring the choroidal thickness in school-aged children could be one of the limitations in our study. The resolution of chorio-scleral boundary could be limited even with the use of enhanced depth imaging in the spectral-domain OCT, especially in those school-aged children with thick choroid. The limited resolution of OCT images captured by the spectral-domain OCT might also limit the accurate prediction of axial elongation by using the choroidal thickness changes. To improve the visualization of the chorio-scleral boundary and detect detailed changes in choroidal thickness, swept-source OCT that utilizes a longer wavelength (1050 nm compared to 870 nm in spectral-domain OCT) could be used in the future studies [43]. Another limitation of the study was the absence of information on short-term responses of the choroid to DIMS lenses within an hour. The current findings solely revealed long-term changes in choroidal thickness induced by DIMS lenses. Investigating the short-term changes in the choroidal thickness to DIMS lenses within an hour may help prove its usefulness in predicting axial elongation after six months of DIMS lenses.

## Conclusions

This is the first study demonstrating that the choroid expanded in response to DIMS lens wear in school-aged children. The choroidal thickening was observed as early as one week of lens wear and was found to be sustained over the long term, which correlates well with the sustained effect of DIMS lens in slowing eye growth. Additionally, the changes in choroidal thickness after three months of lens wear significantly predicted the changes in AL after one year. Our findings highlight the potential use of early and sustained SfChT changes as a predictor of treatment efficacy with DIMS lenses for myopia control.

## Data Availability

Not applicable.

## Abbreviations

DIMS: Defocus incorporated multiple segments
SfChT: Subfoveal choroidal thickness
SV: Single vision
AL: Axial length
DISC: Defocus incorporated soft contact
OCT: Optical coherence tomography
SER: Spherical equivalent refractive errors
SD: Standard deviation
SEM: Standard error of the mean
